# Mycotoxin management in Sub-Saharan Africa: A comprehensive systematic review of policies and strategies in Malawi

**DOI:** 10.1016/j.toxrep.2024.101871

**Published:** 2024-12-18

**Authors:** Chimwemwe Chilenga, Kingsley Masamba, William Kasapila, Brown Ndhlovu, Victor Munkhuwa, Lintle Rafoneke, Kennedy Machira

**Affiliations:** a Lilongwe University of Agriculture and Natural Resources, Bunda College Campus, Department of Food Science and Technology, P.O. Box 219, Lilongwe, Malawi; b Department of Nutrition, HIV and AIDs, Ministry of Health, P/Bag B401, Lilongwe, Malawi; c Browns Consulting Company PO Box 274, Rumphi, Malawi; d Ministry of Health, Lilongwe Health Office, PO Box 1274, Lilongwe, Malawi; e Department of Agricultural Economics, LUANAR, Africa Center of Excellence in Agriculture Policy Analysis (APA), P.O box 219, Lilongwe, Malawi

**Keywords:** Malawi, Policies, Health, Agriculture, Trade, Environmental, Nutrition

## Abstract

Food safety challenges, such as mycotoxin contamination, pose severe threats to public health, agricultural productivity, and economic development across Sub-Saharan African countries and beyond. This study investigated whether government policies related to food safety adequately address these concerns, using Malawi as a case study. We systematically reviewed 29 government-authored policy documents related to food safety. These documents were categorized into six sectors: Agriculture, Environment, Nutrition, Health, Trade and Industry, and Education. Our analysis revealed critical gaps in addressing mycotoxin concerns in these policies, with only 4 of the 29 policy documents (14 %) addressing food safety and mycotoxin management. In contrast, 13 policy documents (45 %) did not address these issues at all, while 12 policy documents (41 %) focused solely on food safety management without addressing mycotoxin contamination. Notably, Malawi's long-term development blueprint, ***Malawi 2063***, does not include mycotoxin management, underscoring a critical policy gap and broader systemic challenges in integrating food safety and mycotoxin control into national frameworks. Furthermore, Malawi lacks a dedicated sector responsible for food safety and a comprehensive national food safety policy to coordinate efforts in mycotoxin control. While this study centers on Malawi, the findings resonate globally, particularly in Sub-Saharan Africa and other countries with similar agricultural and economic contexts. Addressing these systemic policy gaps is vital for developing integrated food safety frameworks that combat mycotoxin contamination, strengthen sustainable food systems, enhance public health, and foster economic resilience. These findings also provide a replicable model for policy analysis, contributing to international discourse by emphasizing the importance of aligning food safety governance with global development priorities, such as the Sustainable Development Goals.

## Highlights

• This study examines how Malawi's government food policies address food safety and mycotoxin contamination issues.
• Twenty-nine policy documents from different sectors were analyzed to determine if they address food safety and mycotoxin contamination.
• Only 4 of 29 policies (14 %) addressed both food safety and mycotoxin while 45 % (13 ) addressed neither food safety nor mycotoxin.
• Malawi lacks a National Food Safety Policy and a dedicated food safety sector.
• Malawi 2063, the country's long-term development framework lacks provisions for mycotoxin control indicating a policy gap.

## Graphical Abstract

**Figure:**
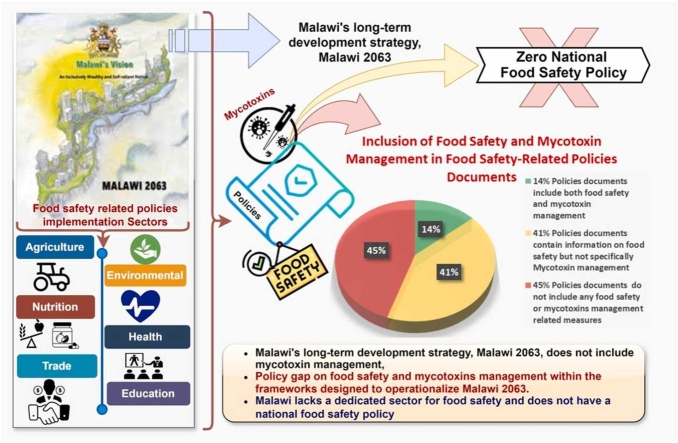

## 1 Introduction

Despite significant advancements in agricultural production and food safety technologies, mycotoxin contamination continues to pose a persistent and serious threat to global food security. These toxic compounds, produced by fungi as secondary metabolites, contaminate a wide range of agricultural products, leading to severe food safety and nutritional issues. Mycotoxins can result in acute and chronic poisoning in both humans and livestock, significantly impacting public health and economic stability [47], [55]. These mycotoxins proliferate optimally in temperatures ranging from 10 to 45 ºC, with moisture levels above 9 % and relative humidity between 65 % and 90 %, conditions commonly found in sub-Saharan Africa (SSA) countries [4], [38], [47]. The absence or inadequacy of food safety regulations, inappropriate farming and storage practices, limited mycotoxin detection mechanisms, resource constraints, and low awareness exacerbate the problem. Additionally, unharmonized policy frameworks combined with ineffective enforcement mechanisms and infrastructure further complicate the issue [21], [31], [32], [37].

Globally, over 4.5 billion people, predominantly in developing nations like Malawi, are chronically exposed to aflatoxin-contaminated foods [17]. Aflatoxin contamination in key staples such as maize and peanuts represents a significant public health concern and a major barrier to meeting international trade standards. This issue undermines economic growth, hinders food safety, and presents challenges to achieving sustainable food security; a state in which all individuals have access to sufficient, safe, and nutritious food for an active and healthy life [16], [59]. Addressing these challenges is critical, as unsafe food impedes progress on some Sustainable Development Goals (SDGs), particularly those related to health (SDG 3), food security (SDG 2), and poverty reduction (SDG 1) [20], [37].

**Fig. 1:**
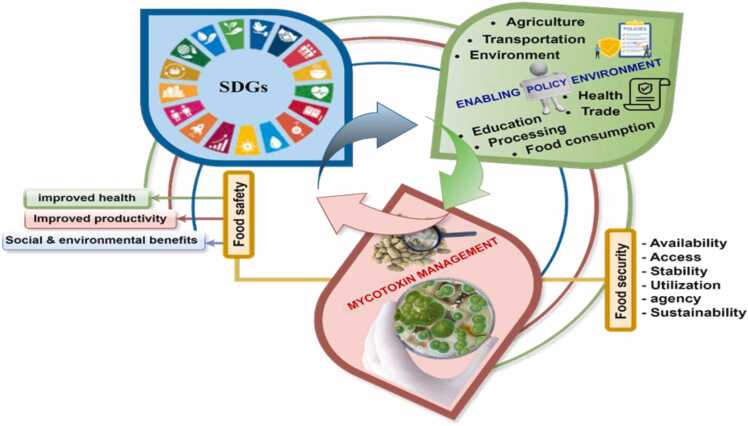
Viscous cycle between the attainment of policy goals and mycotoxin management.

Integrating mycotoxin management into food value chain policies and development frameworks can enable effective mitigation strategies, whereas its absence undermines the achievement of key policies and developmental goals [16], [37]. For instance, achieving food security and zero hunger, as outlined by the Sustainable Development Goals (SDGs), requires that available food is both safe and sufficient to meet the physiological needs of each individual [26], [59], [61], [62], [8]. Food safety and security are closely linked to various economic, social, and environmental aspects of well-being, reflecting their interconnectedness with at least six SDGs [16], [20], [37]. In summary, the attainment of policy goals, food safety, and SDGs are intricately linked. Mycotoxin-related challenges impede the achievement of SDGs and policies, while effective policies are crucial for enhancing food safety interventions. For details on the vicious cycle between the attainment of policy goals, mycotoxin management, and food safety, refer to Fig. 1.

Although specific aflatoxin standards exist in Malawi, such as those outlined in the Malawi Bureau of Standards for peanuts and peanut butter [33], enforcement remains limited. As a result, the prevalence of aflatoxin contamination remains alarmingly high even in groundnuts, a product that is ostensibly covered by these regulatory standards [11], [30], [41], [42], [44]. Consequently, Malawi has incurred substantial economic losses, estimated at USD 392.6 million annually, due to aflatoxin exposure alone [48] reflecting the need for more coordinated and comprehensive efforts in mycotoxin control, beyond merely incorporating standards into regulatory frameworks [37], [39], [48]. This challenge is not unique to Malawi; many other sub-Saharan African (SSA) countries also face similar enforcement limitations, where aflatoxin regulations are either poorly implemented or selectively applied, often focusing solely on export markets while neglecting domestic food safety trade [4], [34], [39]. Unlike countries with robust food safety frameworks, such as Ghana and Kenya, Malawi lacks a comprehensive National Food Safety Policy to coordinate multi-sectoral interventions and address critical points of contamination.

Most published reviews on mycotoxin contamination focus on their prevalence, impact on humans and livestock, and regulations in SSA countries [21], [23], [3], [4], [36], [45]. Nevertheless, there is limited literature on the coverage of mycotoxin management in policy documents and developmental frameworks, particularly in Malawi. This study therefore aimed at systematically analyzing food safety-related policy documents across Malawi's food value chain, focusing on the extent to which mycotoxin management is addressed across key sectors. By offering an in-depth review of government-authored policies, this study provides actionable recommendations aimed at improving food safety governance. The work presents a comprehensive, cross-sectoral policy assessment, emphasizing the importance of integrated mycotoxin management across agriculture, nutrition, health, and trade sectors. Additionally, the study links policy deficiencies directly to the economic burden of aflatoxin contamination in Malawi, a perspective not widely explored in previous studies. In addressing these critical gaps, this study contributes to the literature on mycotoxin management and food safety governance in Malawi.

## 2 Methods and materials

### 2.1 Search strategy

This study employed a systematic review of sector-specific policy documents, a methodology rarely used to evaluate the alignment of policies with food safety and mycotoxin management. To find relevant policies for the study, we followed a series of steps. First, we conducted an electronic web-based search using specific keywords such as 'Malawi', 'policies', 'health', 'agriculture', 'trade', 'environmental', and 'nutrition'. We used different websites and links to retrieve various food safety-related policy documents across the food value chain. Secondly, we contacted individuals from concerned ministries and asked for policies in use, under review, or expired in the past two years. Thirdly, we excluded policy documents that did not meet our inclusion criteria. Thereafter, we aligned the included policy documents towards the food value chain. All other policies that could not align properly along the food value chain were removed. Finally, we downloaded all policy documents that met our inclusion criteria into one folder.

### 2.2 Inclusion and exclusion criteria

This study focused on Malawi Government-authored policy documents that have been in use, under review or expired within the last two years. Legal documents such as Bills and Acts were excluded from this study. Additionally, policy documents that expired more than two years ago, those developed by government partners and other stakeholders, and those published after March 1, 2024, were not considered. Furthermore, Malawi 2063 [14] and Malawi 2063 first 10 years' plan (MIP-1) [15] were included. All policy documents included in this study were related to one or several stages of the food value chain. Policies from other sub-Saharan African countries, other than Malawi, were not included.

### 2.3 Data extraction

The main objective of the data extraction process was to determine if the included policies contained information related to food safety and specifically mycotoxin contamination. Additionally, the data extraction focused on identifying policies related to specific food value chain processes such as production, storage, processing, transportation, marketing, and consumption. Other information such as the sector of the policy, title, expiry date, policy goal, policy outcomes, objectives related to food safety and mycotoxins, priority areas, performance indicators, and targets were systematically extracted. To collect this data, we created a standardized Excel sheet to record the extracted information. We critically analyzed and evaluated policy indicators and targets, assigning values as follows: zero (0) indicated that the policy document did not address food safety or mycotoxin contamination, one (1) indicated that the policy document contained information on food safety but did not specifically mention mycotoxin, and two (2) was assigned when the policy document addressed both food safety and mycotoxin contamination.

## 3 Results and discussion

### 3.1 Description of data

**Fig. 2:**
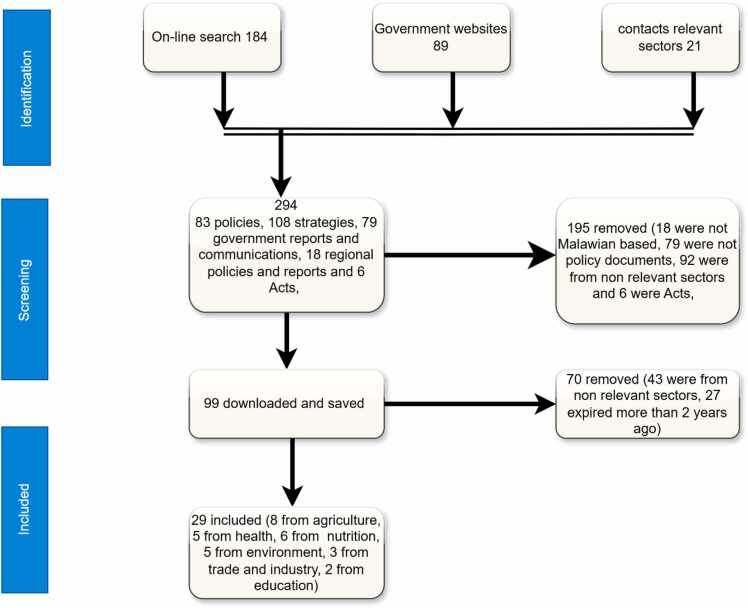
Summary of the screening process to identify policies for the review.

The search yielded a total of 294 documents, from various government sectors. These documents included policies, strategies, government communications, and reports. However, out of these, only 99 potential documents were downloaded with distribution across sectors as follows: 35 from the health sector, 27 from agriculture, 17 from nutrition, 9 from the environmental sector, 4 from trade and industry, 4 from education, 2 from information, and 1 from transport. After careful analysis, we found that only 29 policy documents satisfied our inclusion criteria, including 8 from the Ministry of Agriculture, 6 from the Department of Nutrition, 5 from the Ministry of Health, 5 from the Environment, 3 from the Ministry of Trade and Industry, and 2 from the Ministry of Education. It is worth noting that we did not include any policy specifically addressing food transportation. The documents included 15 policies, 13 strategies, and 1 implementation plan. For more details on the included and excluded policy documents at different screening stages, please refer to Fig. 2.

Out of the 29 policy documents identified as directly related to food safety issues, only 4 addressed both food safety and mycotoxin management, representing just 14 % of all food safety-related policy documents. In contrast, 12 policy documents (41 %), exclusively addressed food safety without including mycotoxin management. It is disconcerting that nearly 45 % (13 policy documents) do not address food safety or mycotoxin management. Furthermore, most policies that mention food safety and mycotoxin management do so inadequately.

**Fig. 3:**
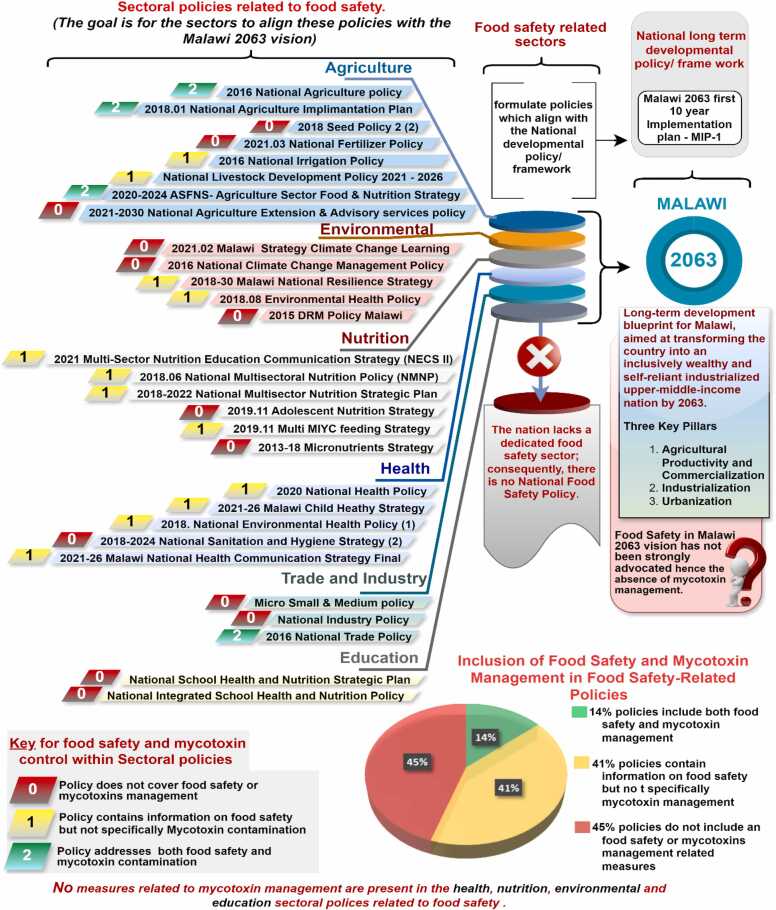
Coverage of food safety and Mycotoxin contamination in different policy documents in Malawi.

It is important to note that Malawi does not have a food safety policy and lacks a dedicated sector for food safety. In countries with established national food safety policies, such as Ghana (Republic of [10]), Kenya (Republic of [24]), and Nigeria (Republic of [43]), there is a coordinated integration of food-related policies across relevant ministries and sectors, including health, agriculture, trade, environment, education, and local government. This comprehensive approach facilitates effective food safety management and ensures a holistic implementation of safety standards**.** Refer to Fig. 3 below for a detailed illustration of the coverage and gaps in food safety and mycotoxin management within all food safety-related policy documents in Malawi.

The information in Fig. 3 above reflects only those policy documents that are hypothetically supposed to address food safety and mycotoxin management. The documents presented, have been grouped into six sectors Agriculture, Environmental, Nutrition, Health, Trade and Industry, and Education based on their origin. These documents have further been classified into three categories (0, 1, and 2) depending on their coverage of food safety and mycotoxin management. Category 0 includes policy documents that do not contain information or measures directly aimed at addressing food safety or mycotoxin management. Category 1 comprises policy documents that exclusively address food safety without covering mycotoxin management. Category 2 encompasses policy documents that address both food safety and mycotoxin management.

### 3.2 Food safety and mycotoxin control issues in Malawi’s vision 2063 and Malawi 2063 first 10-year implementation plan (MIP-1)

Malawi aspires to transform into an industrialized upper-middle-income country by 2063, guided by a comprehensive long-term policy framework referred to as Malawi 2063 [14]. This vision hinges on the strategic enhancement of the agricultural and mining sectors, recognizing their critical contributions to economic growth and sustainable development. The framework envisages moving beyond the current production of maize and a few cash crops mainly for food security. Instead, Malawi aims at diversifying agricultural production by emphasizing high-value crops that meet global health standards and are resilient to environmental shocks. Additionally, the framework envisions an agricultural sector that provides access to nutritious foods, especially in the first 1000 days of life to eliminate stunting and improve cognitive development. Furthermore, the framework seeks to halt intergenerational stunting by prioritizing women’s health and nutrition and promoting access to quality and diversified diets among young children and pregnant women as stipulated in the Scaling up Nutrition (SUN) 1000 global movement [60].

Although the Malawi 2063 framework envisages an effervescent agricultural sector that feeds into prolific manufacturing, industrialization, and exports that meet global health standards, the findings of this study highlight a significant oversight in this vision. The framework does not explicitly address mycotoxins or food safety, which are among the major impediments to agricultural exports. The Malawi blueprint is silent on how the country will achieve high-value agricultural exports. Currently, Malawi is one of the SSA countries that lost the overseas foreign market for agricultural produce because they could not meet the regulatory standards such as aflatoxin acceptable limits [39], [58]. Consequently, the country shifted to the informal regional market that pays less and does not generate tax revenue for the government [39]. Achieving safe foods that meet mycotoxin acceptable limits is crucial for Malawi to achieve its goal of increasing revenue from agricultural exports.

Malawi has set the ambitious goal of eradicating stunting by the year 2063. However, the presence of mycotoxins, which have been linked to stunting [54], poses a significant challenge to achieving this target, especially since 60–80 % of food crops are affected by mycotoxins [6]. In the current agenda 2063 framework, the country envisages achieving a healthy population and an increase in life expectancy. However, the current mycotoxin contamination levels in the food value chain, challenge the achievement of this vision. Studies have linked mycotoxin contamination to various health issues such as infertility [25], [46], congenital disorders [9], gastrointestinal problems [2], [27], kidney disorders [22] carcinogenicity [22], [5], liver damage [51], [52] immunosuppression [28], [56], cardiac impairment [50], cytotoxicity [49], [53], genotoxicity [57], and neurotoxicity [22]. Moreover, the health of the human population is heavily determined by the quality of foods and food-producing ecosystems. In summary, the attainment of many indicators outlined in Malawi's 2063 developmental agenda could be compromised due to the absence of explicit provisions addressing food safety and mycotoxin control. Furthermore, Chilaka and his colleagues [4] argue that food safety issues are inter-sectoral problems, occurring at the interface of health, agriculture, trade, and industry. They may be systematically under-estimated but their effects have a long-lasting impact on the country’s progress.

### 3.3 Mycotoxin control issues in different sector policies

#### 3.3.1 Agriculture sector policies

The Malawian economy is heavily dependent on agriculture, with about 77 % of the population relying on this sector for their livelihoods [7]. Recognizing agriculture's vital role, the Government of Malawi developed the National Agriculture Policy (NAP), as a cornerstone of agricultural governance. The NAP is implemented through the National Agricultural Investment Plan (NAIP). Government of Malawi [13] and other policy documents across various agricultural departments. The NAP aims to achieve a sustainable agricultural transformation that will result in significant growth of the agricultural sector, increased incomes for farming households, and improved food and nutrition security for all Malawians. However, mycotoxin contamination poses a significant challenge to the NAP's objectives. Mycotoxin contamination is associated with low agricultural production; both crops and livestock [40], diminishing the quality, quantity, and market value of agricultural products, thereby reducing farmer incomes in regions with prevalent contamination [39]. Alarmingly, eight agriculture-related policy documents reviewed in this study offer limited consideration of food safety and mycotoxin control. Only three documents, the National Agricultural Policy (Ministry of Agriculture, 2016), National Agricultural Investment Plan [13] and the Agricultural Sector Food and Nutrition Strategy (Ministry of [1]) address both food safety and mycotoxin contamination.

The lack of emphasis on mycotoxins in critical policy documents, such as the National Seed Policy [13], National Fertilizer Policy [29], and National Agricultural Extension and Advisory Policy [35], underscores a gap in Malawi's agricultural policy framework. Integrating mycotoxin management into these policy documents could significantly reduce contamination risks across the food value chain. Despite the National Agriculture Policy's emphasis on improving food security and nutrition, the absence of mycotoxin management in the sector’s policy documents undermines progress toward achieving this objective. This gap is especially troubling in the National Livestock Development Policy, which neglects to address the risks posed by mycotoxins in livestock production.

It is important to note that food security cannot be achieved without food safety. The relationship between food safety and food security is often characterized as a vicious cycle where compromised food safety can lead to foodborne illnesses, affecting consumers' health and well-being, and reducing their ability to work, earn income and contribute to food security. In many developing countries, food security is undermined as individuals struggle to access safe and nutritious food, perpetuating a cycle of poverty and malnutrition. In countries such as Malawi, where food security is already precarious, people may overlook food safety standards to ensure immediate access to food, exacerbating food safety risks. This self-perpetuating cycle, where compromised food safety contributes to ongoing health challenges, economic instabilities, and food insecurity, can be halted by integrating food safety and mycotoxin management into food security initiatives. Addressing the multifaceted nature of mycotoxins requires a comprehensive approach at all levels [4]. Ultimately, integrating mycotoxin control into these policies is essential for sustainable growth in agriculture, improving farm incomes, and ensuring nutrition security.

#### 3.3.2 Health sector policies

The health effects of mycotoxins are extensive and encompass a range of severe conditions, including carcinogenic, cytotoxic, mutagenic, genotoxic, teratogenic, hepatotoxic, nephrotoxic, neurotoxic, and immunotoxin effects. These adverse health impacts significantly lower the quality of life, particularly for vulnerable populations such as children, pregnant women, and individuals with HIV/AIDS, whose compromised immune systems heighten their susceptibility to mycotoxin-related complications. Despite these severe consequences, mycotoxin contamination remains under-addressed within Malawi’s health sector policies, which limits opportunities for a coordinated public health response.

In this study, five health sector policy documents were examined to assess their coverage of food safety and mycotoxin contamination. The review revealed that none of these documents explicitly recognized mycotoxin contamination as a public health issue. Four of the policy documents (National Health Policy, National Health Communication Strategy 2021 – 2026, Malawi Child Health Strategy II 2021 – 2026, and National Environmental Health Policy, 2018) mentioned food safety only, without addressing specific contaminants or their health implications. The National Sanitation and Hygiene Strategy [13], a key policy framework for improving public health outcomes, omitted any reference to food safety or mycotoxins entirely, further underscoring the lack of integration between sanitation, hygiene, and food safety efforts.

This gap is particularly concerning, given the shared goal of all health-related policy documents to achieve Universal Health Coverage (UHC), which seeks to ensure equitable access to essential health services. Mycotoxin contamination, as a significant contributor to both acute and chronic illnesses, directly threatens the realization of UHC. The lack of specific mycotoxin control measures within health sector policies represents a missed opportunity to reduce the prevalence of foodborne illnesses and improve public health outcomes. Moreover, this oversight underscores a critical disconnect between the health and agricultural sectors. While agricultural policies often emphasize mycotoxin standards for export and trade, the health implications of mycotoxin exposure for domestic populations are largely neglected. This misalignment limits the effectiveness of food safety initiatives and reduces their potential to safeguard public health. By addressing this gap, Malawi can strengthen its commitment to universal health coverage while protecting its population from the harmful effects of mycotoxins.

#### 3.3.3 Nutrition-related policies

Studies have linked chronic, suboptimal exposure to mycotoxins with stunted growth in children [54]. Despite this, none of the six nutrition-related policy documents examined (National Micronutrient Strategy NMS for Malawi 2013 - 2018, National Multi-Sector Nutrition Policy 2018 – 2022, National Multisector Nutrition Strategic Plan 2018–2022, Multi-Sectoral Adolescent Nutrition Strategy 2019–2023, Multi-Sector Maternal, Infant and Young Child Nutrition Strategy 2019–2023, and Multi-Sector Nutrition Education and Communication Strategy (NECS) II 2021–2025) identify mycotoxins as a concern. All the nutritional sector policy documents aim at eradicating all forms of malnutrition, yet they overlook the critical association between mycotoxins and stunted growth in children. While the nutritional quality and safety of food are inherently linked, the prioritization of donor-driven interventions targeting stunting and malnutrition has channeled substantial resources toward the nutrition sector, potentially overshadowing essential food safety efforts [39]. This neglect is particularly troubling, given the substantial effects of mycotoxins on nutrition and health outcomes. The omission of mycotoxin management from these nutrition-related policy documents is yet another missed opportunity in the broader fight against mycotoxin contamination. By not acknowledging the role of mycotoxins in contributing to stunted growth and other nutritional outcomes, these policies fall short of offering a holistic solution to malnutrition in all its forms. Integrating mycotoxin control measures into nutrition policies is essential for developing comprehensive strategies that address the detrimental effects of mycotoxins on nutrition, thus advancing efforts to eradicate malnutrition in all its forms. Additionally, incorporating mycotoxin management into nutrition policies would help bridge the existing gap between food safety and nutrition efforts, ensuring a more unified and effective approach to safeguarding public health.

#### 3.3.4 Trade and industry policies

Mycotoxins have become a critical issue in international trade, prompting increased attention to their presence in agricultural products in global markets due to their significant health concerns [39]. The Malawi National Trade Policy seeks to make the country a globally competitive export-oriented economy, generating higher and sustainable livelihoods through trade. The policy aims to boost exports by improving domestic market structures and integrating into regional and global markets through value chains. This aligns with the second pillar of the Malawi Vision 2063, which aims to transform the economy from a predominantly importing to an industrialized exporting economy [14]. However, countries across the globe have imposed stringent regulations regarding mycotoxin levels in imported goods to safeguard public health leading to heightened scrutiny and rejection of contaminated products exceeding acceptable thresholds at borders. Malawi and several other SSA countries lost their overseas market for different agricultural products due to elevated aflatoxin contamination levels [4], [39]. However, of the three policy documents from the Ministry of Trade and Industry that were examined, only the National Trade Policy recognizes mycotoxins as a challenge. It is discouraging to note that the other two policy documents namely the National Industry Policy and the Micro Small and Medium Enterprise Policy [18] do not address food safety or mycotoxin as a challenge.

The National Industry Policy specifically seeks to reshape Malawi’s economic landscape by increasing the proportion of manufacturing in the country’s Gross Domestic Product (GDP), aligning with the industrialization pillar of Malawi Vision 2063. However, it is notable that this policy fails to address food safety and mycotoxin issues, despite the significant implications these have for trade and economic growth. While the National Industrial Policy reflects Malawi's ambitions for advancing manufacturing and economic expansion, its disregard for critical food safety and mycotoxin concerns could impede its successful implementation, especially in agro-based industries. Without integrating safeguards against these health risks and productivity constraints, the attainment of industrialization goals may be compromised, particularly as export rejections from international markets can result from non-compliance with stringent food safety standards.

It is worth noting that Malawi lacks a dedicated occupational health and safety policy. Mycotoxin exposure is an occupational hazard for workers in agriculture, food processing, and storage. These workers are at heightened risk of inhaling mycotoxin-contaminated dust, leading to serious health issues, including respiratory ailments and immune suppression. Workplace safety concerns are addressed through the Occupational Safety, Health, and Welfare Act (OSHWA) [19]. However, this Act does not equally explicitly address mycotoxins risks. Integrating mycotoxin control into OSHWA would be a proactive step toward safeguarding workers’ health and reducing productivity losses.

Incorporating mycotoxin management and food safety measures into trade and industry policy frameworks is crucial to achieving sustainable, and inclusive industrial development in Malawi. An integrated approach would strengthen trade resilience, ensure compliance with international standards, and bolster Malawi’s export competitiveness by mitigating the health and economic risks posed by mycotoxins. By addressing mycotoxin contamination, Malawi can better meet international food safety standards, enabling access to higher-value export markets and enhancing economic growth.

#### 3.3.5 Environmental policies

The complex interplay between environmental conditions and food safety, particularly in relation to mycotoxin prevalence along the food value chain, underscores the urgent need to address environmental factors as part of food safety strategies [63]. Environmental conditions, especially during storage, are pivotal in influencing the degree of mycotoxin contamination. In this study, five policy documents from the environmental sector were examined. Alarmingly, none of these documents identifies mycotoxins as a potential area of concern, despite the well-established link between environmental factors and mycotoxin contamination. More concerningly, three out of these five documents omit food safety issues altogether, while the Environmental Health Policy [13] and the Malawi National Resilient Strategy only address food safety without mention of mycotoxins specifically.

The National Environmental Policy seeks to achieve the highest possible level of health and well-being for all Malawians by reducing morbidity and mortality associated with environmental health risks. Food safety and hygiene are recognized as priority areas within this policy, which includes aspirations for a functional National Food Safety and Quality Control system. Although the policy lacks specific strategies for mycotoxin mitigation, the incorporation of food safety considerations represents a foundational step towards fostering sustainable food security, safeguarding public health, and advancing economic growth through healthier food systems.

Furthermore, food safety and mycotoxin management are conspicuously absent from the National Disaster Risk Management Policy [12]. This policy aims to reduce disaster-related losses of life as well as social, economic, and environmental assets for individuals, communities, and the nation. However, despite the heightened vulnerability of communities during disasters, where risks of food spoilage and contamination are often elevated, the policy does not include specific food safety protections. This oversight potentially exposes communities to health hazards during disaster recovery periods, highlighting the critical need for disaster management policies that comprehensively integrate food safety considerations to protect public health during and after crises.

#### 3.3.6 Education policies

An examination of two policy documents from the Ministry of Education, the National Integrated School Health and Nutrition Policy and the National School Health and Nutrition Strategic Plan reveals a concerning gap: neither addresses food safety issues or the risk of mycotoxin contamination. These policies are designed to support the health and nutritional well-being of school-aged children, yet they overlook essential food safety considerations that are crucial for achieving this goal. The absence of measures addressing food safety and mycotoxins is particularly troubling given the heightened vulnerability of children to the adverse effects of contaminated food, as well as the potential for long-term health consequences.

Mycotoxins, for example, pose significant health risks that can profoundly impact child development. Chronic exposure to mycotoxins has been linked to stunted growth, weakened immune systems, and impaired cognitive function, effects that can hinder academic performance and overall well-being. Without adequate safeguards to ensure that school meals are free from contaminants, there is a missed opportunity to provide children with a truly supportive nutritional environment. Moreover, children in Malawi, where food security challenges often lead to a reliance on potentially contaminated staple foods, may be at higher risk of chronic mycotoxin exposure, which further underscores the importance of integrating food safety protocols into school health policies.

Integrating food safety measures, specifically mycotoxin management, would bolster these policies by not only protecting children’s health but also enhancing their ability to thrive in educational settings. Healthier children experience fewer illness-related absences and are more likely to benefit from academic opportunities, resulting in better educational attainment. The absence of mycotoxin management strategies in school health policies not only diminishes the protective scope of these programs but also risks undermining the broader goals of educational equity and youth empowerment. Therefore, a holistic approach that integrates food safety into school health and nutrition policies is essential. Such an approach would help ensure that children receive not only the nutritional but also the health benefits needed to excel in their learning environments and contribute meaningfully to Malawi’s future development trajectory.

## 4 Conclusion

In conclusion, the analysis of policy documents spanning Malawi's food value chain reveals a significant and troubling gap in addressing food safety and mycotoxin contamination. Despite the well-documented health risks of mycotoxin exposure, including its carcinogenic effects and its contribution to childhood stunting, policies governing food production, health, and consumption largely neglect mycotoxin control. This omission is particularly evident in nutrition policies, which fail to acknowledge the critical role of mycotoxin exposure in undermining nutritional outcomes and exacerbating malnutrition. In the health sector, policies fail to recognize mycotoxin contamination as a critical public health issue, despite its extensive adverse effects on vulnerable populations, including children, pregnant women, and individuals with compromised immune systems. These deficiencies not only limit the country’s ability to reduce the prevalence of foodborne illnesses but also hinder progress toward universal health coverage by exacerbating malnutrition and straining healthcare systems. Additionally, the absence of explicit food safety and mycotoxin control measures within the Malawi 2063 agenda highlights a systemic failure to prioritize these issues within the country’s development framework.

Other key challenges include the lack of a dedicated National Food Safety Policy, unclear mandates for food safety oversight, and poor coordination among stakeholders. While some specific standards exist, such as those regulating aflatoxin levels in groundnuts, their enforcement is weak due to a lack of supportive and comprehensive policy mechanisms. These systemic gaps hinder progress toward improving food safety governance and addressing the multifaceted impacts of mycotoxins on health, nutrition, and economic development. The findings underscore the urgent need for a comprehensive mycotoxin control program that integrates food safety, nutrition, health, and economic considerations. Embedding mycotoxin management strategies into existing policy frameworks, alongside strengthened regulatory enforcement, and capacity-building efforts, could enable Malawi to achieve significant advancements in public health protection, food security, and sustainable economic growth. The following actionable recommendations are therefore proposed:

1. Develop a National Food Safety Policy – Establish a comprehensive framework to coordinate food safety efforts, including explicit strategies for mycotoxin management across the food value chain.
2. Strengthen Regulatory Enforcement – Invest in enforcement mechanisms for existing standards, such as aflatoxin thresholds, by providing adequate funding, training for regulatory bodies, and modern testing infrastructure.
3. Integrate Mycotoxin Control into Development Agendas – Embed specific mycotoxin management strategies into national development frameworks, including the Malawi 2063 agenda, to align food safety with broader goals such as improved health and economic development.
4. Foster Stakeholder Collaboration – Create multi-sectoral platforms that bring together government agencies, academia, private sector actors, and international partners to coordinate food safety interventions.
5. Build Research and Surveillance Capacity – Strengthen research initiatives and surveillance systems to monitor mycotoxin prevalence, impacts, and the effectiveness of mitigation measures, ensuring data-driven policy-making.

This work uniquely emphasizes the intersection of food safety, nutrition, and economic development within the context of mycotoxin management and the suggested actionable recommendations can enhance food safety governance in Malawi. While this analysis is specific to Malawi, the challenges and gaps identified likely reflect broader trends across sub-Saharan Africa. Coordinated regional initiatives are vital to addressing mycotoxin contamination as a critical issue, fostering collaborative frameworks to mitigate its adverse effects on health, economic productivity, and food security. Such efforts would not only strengthen regional food safety systems but also contribute to achieving shared development objectives, including improved nutrition, universal health coverage, and sustainable economic growth.

## Declaration of Competing Interest

The authors declare that they have no known competing financial interests or personal relationships that could have appeared to influence the work reported in this paper. The authors have no conflict of interest

## Data Availability

No data was used for the research described in the article.
